# Impact of implementation interventions to improve substance use service delivery on recidivism among justice-involved youth

**DOI:** 10.1186/s40352-023-00210-3

**Published:** 2023-02-28

**Authors:** Angela A. Robertson, Sheena Gardner, Richard Dembo, Michael Dennis, Jennifer Pankow, Khirsten J. Wilson

**Affiliations:** 1 Social Science Research Center, Mississippi State University, 1 Research Blvd., Suite 103, Starkville, MS 39759 USA; 2 Department of Criminology, College of Behavioral & Criminal Sciences, University of South Florida, Tampa, FL USA; 3 Lighthouse Institute, Chestnut Health Systems, Normal, IL USA; 4 Institute of Behavioral Research, Texas Christian University, Fort Worth, TX USA

**Keywords:** recidivism, substance use treatment, behavioral health, organizational interventions, implementation strategies

## Abstract

Substance use disorders (SUD) are prevalent among justice-involved youth (JIY) and are a robust predictor of re-offending. Only a fraction of JIY with substance use problems receive treatment. This paper describes the impacts of system-level efforts to improve identification and referral to treatment on recidivism of JIY. A cluster randomized trial involving 20 county juvenile justice agency sites across 5 states was used to implement an organizational intervention (Core vs Enhanced) to juvenile justice staff and community-based treatment providers, working with 18,698 JIY from March 2014 to August 2017. Recidivism rates over four study time periods were examined. Logistic regression was used to predict recidivism as a function of site, need for SUD services, level of supervision, time, organizational intervention, and time x intervention interaction terms. Results indicated that Enhanced sites showed decreased levels of recidivism compared to Core-only sites, where it increased over time. Additionally, need for SU services, level of supervision, and site were significant predictors of reoffending. Findings suggest the potential value of facilitation of juvenile justice agency efforts to increasing identification of and referral to SUD services of JIY in need of such services for reducing further contact with the legal system.

## Background

Reducing recidivism among youth offenders is a commonly held goal across juvenile justice (JJ) systems (Schweitzer et al., 2017). Examining individual risk factors for youth involved with the legal system is essential in reducing the likelihood of reoffending. Substance use (SU) is among the factors routinely assessed at intake to the JJ system in reviewing recidivism risk (Baglivio, 2009) because of its strong association with delinquent behavior (DeLisi et al., 2015; Dembo et al., 1997; Leukefeld et al., 2017; Mulvey et al., 2010), and recidivism (Hoeve, McReynolds, & Wasserman, 2014; McReynolds et al., 2010; Robertson et al., 2020; Stoolmiller & Blechman, 2005; Van der Put et al., 2014). Youth in the juvenile justice system have substantially higher rates of alcohol, marijuana, and cocaine use compared with youth in the general population (Miech et al., 2018; Mieczkowski et al., 1998; Mulvey et al., 2010), with approximately 51% of youth involved with community supervision having SU problems requiring treatment (Scott et al., 2019). Screening for SU disorders at intake to the JJ system is necessary to determine the need for treatment services since untreated SU disorders and mental health problems among justice-involved youth increases the likelihood of continued contact with the legal/justice systems (Cuellar et al., 2006; Hoeve, McReynolds, and Wasserman, 2013), as well as escalates the severity of offending behavior (Hoeve, McReynolds, McMillan, and Wasserman, 2013).

To effectively address SU among juvenile offenders and potentially reduce recidivism, JJ agencies should identify youth with SU problems, actively link them to appropriate services, and ensure that they are retained in treatment for a sufficient period to achieve clinical improvement (Belenko et al., 2017). Ideally, youth would receive an initial screening at intake to the JJ system and, if warranted by screening results, receive a more in-depth clinical assessment to determine need of services. Probation officers are key actors in the screening and referral process as they are privy to information (e.g., youth histories and symptomology) that would allow them to appropriately recommend mental health and SU services for justice-involved youth (Stiffman et al., 2004).

Although receipt of treatment reduces recidivism (Haerle, 2016; Schweitzer et al., 2017), several factors can affect probation officer referrals. Probation officers with higher levels of mental health competence are better able to identify mental health needs and refer youth to services (Stiffman et al., 2004; Wasserman et al., 2008). Officers holding stigmas towards mental health (Louden et al., 2018) or those experiencing conditions such as burnout may be less engaged in the delivery of services (Salyers et al., 2015; White et al., 2015). Additionally, the limited availability of community-based mental health and SU treatment resources is linked to lower rates of referral, presumably due to probation officers’ perception that identifying needs is unnecessary if, ultimately, resources are unavailable (Wasserman et al., 2008). Consequently, many justice-involved youth in need of services do not access treatment (Wasserman et al., 2009; Belenko et al., 2017). A recent study of youth under community supervision found that 70% were screened, more than half were in need of treatment, but only one fifth of those in need were referred to treatment (Wasserman et al., 2021).

The Juvenile Justice-Translational Research on Interventions for Adolescents in the Legal System (JJ-TRIALS) research cooperative was funded by the National Institute on Drug Abuse to develop and test whether a set of organizational interventions involving staff of JJ and Behavioral Health service provider agencies to improve screening, identification of SU service need, referral, treatment initiation, and treatment engagement among youth under JJ community supervision. The Cooperative included six research centers (Columbia University, Emory University, Mississippi State University, Temple University, Texas Christian University, and University of Kentucky), each working closely with a JJ partner in seven states (Leukefeld et al., 2017), a coordinating center (Chestnut Health Systems), an independent Chair of the Steering Committee (George Mason University) and a scientific officer from the National Institute on Drug Abuse. The Cooperative developed the Behavioral Health Services Cascade (hereafter referred to as “Cascade”) framework to facilitate research and practice regarding the best strategies to identify and address SU treatment need within and across agencies, and to document progress toward increasing referral and subsequent service receipt (Belenko et al., 2017; Dennis et al., 2019). Recognizing that coordination is required between the juvenile justice and treatment providers to facilitate youth transition across service sectors (Welsh et al., 2021), research centers recruited 36 county JJ agencies and their local community-based behavioral health treatment partners. Participating sites were either county stand-alone juvenile probation departments or youth courts with in-house juvenile probation staff.

The primary research aims of JJ-TRIALS were to evaluate the relative effectiveness of organizational interventions in increasing rates of (a) substance use screening, (b) service need identification, (c) referral of those in need, (d) treatment initiation among those referred, and (e) treatment engagement among those who initiated treatment (i.e., successive steps in the Cascade). Previous research found that rates of screening remained relatively stable over the study period and referral to treatment among youth in need of SU services increased compared to baseline overall, but the enhanced sites showed greater increases in referrals over time (Belenko et al., 2022). Furthermore, there were significant differences in site referral rates (Belenko et al., 2022). Although the JJ-TRIALS study was not designed to directly assess the impact of intervention activities on recidivism, JJ stakeholders requested that the Cooperative collect additional data from the JJ sites to calculate recidivism rates and to evaluate if recidivism rates changed over the course of the study.

The purpose of this study is to determine whether the organizational interventions implemented by the JJ-TRIALS Cooperative to improve SU treatment service delivery to justice-involved youth differentially impacted 1-year recidivism rates. The two organizational interventions were designed to promote system-wide improvement through inter-agency collaboration and data-driven decision making (DDDM; Mandinach, 2012; Marsh et al., 2006). It was expected that participation in the Core set of strategies would result in decreased recidivism rates over time, and that sites receiving Core + Enhanced (hereafter referred to as “Enhanced”) strategies would show greater improvement compared to Core-only intervention sites. It was hypothesized that relative to baseline, the percentage of new arrests or referrals to juvenile court among youth on community supervision for a 1-year period would be lower in the Enhanced sites than in the Core-only intervention sites by the end of the study.

## Methods

### Research design

The research design used in JJ-TRIALS was a delayed-start (a.k.a., step wedge) randomized control trial, with county-level JJ agencies randomly assigned to an intervention condition using a Matched Block design (see Knight et al., 2016). Sites within a state were paired based on the number of youth aged 10–19 in the general population in the county and the total JJ caseload size; the pair were randomized to one of three start dates. The study was divided into discrete phases that were aligned with the EPIS model to monitor implementation achievements (Aarons et al., 2011; Becan et al., 2020): Exploration (E; T1 Baseline), Preparation (P; T2 Pre-Randomization), Implementation (I; T3 Early Experiment, T4 Late Experiment), and Sustainment (S; T5 Maintenance). The baseline period (T1) lasted 9 to 18 months depending on the site; all other study phases were 6-months in duration. During the baseline period, de-identified youth records data related to the Cascade were collected. All JJ sites received the Core intervention (components described in the next section) during the Pre-randomization (T2) phase of the study. Prior to the 12-month experimental phase (T3 and T4), one of the sites within each pair was randomly assigned to the Enhanced intervention while the other remained a Core-only site that received standard technical assistance in response to questions, but otherwise no further assistance. The fifth and final phase of the study, the maintenance phase (T5), consisted only of data collection, including youth record extraction, staff surveys and focus groups with each site to determine whether sites sustained any new practices. All protocols were reviewed and approved by each research center’s Institutional Review Board and by state/local JJ agencies.

### Organizational interventions

All sites received the Core intervention which involved a mix of training and meetings including: (1) leadership and staff orientation meetings, (2) needs assessment/system mapping and site feedback report to reveal service gaps within the Cascade (Belenko et al., 2017), (3) behavioral health training on evidenced-based substance use treatment practices, family engagement strategies, and inter-agency case planning, and (4) data-driven decision making (DDDM) and goal achievement training on use of data to objectively inform policy and practice improvements (Mandinach, 2012; Marsh et al., 2006) (see Fisher et al., 2018 for a detailed description of the development and implementation of goal achievement and DDDM training in the contexts of JJ-TRIALS). All sites were asked to form an interagency workgroup consisting of JJ and behavioral health service provider staff. Each interagency workgroup was expected to use their site feedback report prepared by research staff to develop and implement plans to better identify youth with substance use problems, and link and retain them in appropriate services to achieve clinical improvement. The interagency workgroups had flexibility in the goals they selected (see Becan et al., 2020 for the types of process improvement plans developed by JJ-TRIALS sites).

Sites in the Enhanced intervention received 1 year of external facilitation in which the facilitator worked collaboratively with the interagency workgroup through a series of process improvement cycles organized around the Plan-Do-Study-Act or PDSA method. External facilitators were intensively trained in the provision of facilitation, teamwork and shared responsibility, conflict resolution, and addressing other human facets of workplace and interagency collaborations (The JJTRIALS Cooperative. 2015). External facilitators were responsible for providing personalized instruction on SMART goals (Rubin, 2002) and DDDM. Specifically, they assisted the JJ agency in the use of their Site Feedback Report, Goal Selection worksheet, and other tools to promote data driven decision making and guided agency efforts to identify goals, develop and implement actions plans, monitor progress, and sustain procedural changes.

The external facilitators were expected to spend 15 to 20 hours per month (not including travel) per site in monthly interagency workgroup meetings and calls/meetings with workgroup members between meetings. External facilitation was expected to produce additional group cohesion, coordination, and effectiveness in the selection and pursuit of improvement goals. Since external facilitation of interagency workgroups can support organizational change and the adoption of evidence-based practices (Fixsen et al., 2009; Powell et al., 2011), sites in the Enhanced condition were hypothesized to yield greater improvements than evidenced in counterpart Core-only intervention agencies.

### Sites and sample

Research sites included county youth courts and juvenile probation departments, and all youth resided within the JJ agency county of jurisdiction. Researchers received de-identified case records on all youth referred to 33 JJ sites between March 2014 and November 2017 to track changes in screening, need identification, referrals to treatment, and, for service initiation, engagement, and continuation of treatment (see Dennis et al., 2019 for a description of data abstraction and coding procedures). This study only used youth case records from 20 JJ sites in the five states (i.e., Florida, Georgia, Mississippi, Pennsylvania, and Texas) that provided the additional data required to examine recidivism within a one-year period. The information needed to determine whether a youth recidivated included unique youth identifiers to track juveniles over time, dates of initial and any subsequent arrests/court referrals, charges/reason for referral, and dates of court hearings and case dispositions.

To follow later cohorts of youth for recidivism, data extractions of youth records continued through August 2018. Despite extending data collection, a proportion of youths entering JJ sites during the Lat Experiment (*N* = 1235 of 3855) and Maintenance (*N* = 615 out of 4204) phases were followed for less than 1 year and were exclude from analyses. After excluding youth with less than 1 year at risk for recidivism, the sample size was 18,698 youth referral records (T1 Baseline *N* = 6869; T2 Pre-Randomization *N* = 5153; T3 Early Experiment *N* = 4056; T4 Late Experiment *N* = 2620).

### Measures

*Recidivism* was defined as a new JJ referral or arrest within 12 months of the initial referral/arrest. JJTRIALS researchers worked with participating JJ agencies providing juvenile community supervision, and the agencies typically had access to juvenile court referrals by law enforcement and arrest data, but not adult arrest data. Variations in JJ site data documentation and multiple new arrests/referrals to JJ within weeks of the initial intake for some youth called for standardizing how these conditions were interpreted in the study datasets. Cases where multiple new JJ referrals occurred within 30 days of the initial intake were collapsed so that a recidivism event was counted only if a second JJ referral occurred 30 or more days after JJ intake (Robertson et al., 2020).

Previous research found large site differences in recidivism rates during the baseline period (Robertson et al., 2020). The overall recidivism rate by site at baseline ranged from 6.4% to 68.8%. This previous study, which tested for differences in recidivism rates across sites, included socioeconomic characteristics of the county, characteristics of youth residing in the county, and local juvenile justice policies and procedures. Examination of possible influences on baseline recidivism found that concentrated disadvantage at the county level, rates of SU screening and determination of SU services need by JJ agency, the percentage of youth of color, rates of juvenile property and felony offenses, and the percentage of juveniles placed on more intensive levels of community supervision all varied substantially across the different JJ agencies and were also associated with recidivism (Robertson et al., 2020). To control for site differences, the *Site* variable was dummy coded such that the largest site (*n* = 2638) was the reference category.

We also included *need for SU services* and *level of supervision* as these variables were among the strongest individual-level predictors of recidivism (Robertson et al., 2020). Determination of need for SU services was based on the presence of one or more of the following indicators: referral to the court for alcohol or drug related offenses; results from drug testing, screening tools, and clinical assessments; JJ staff recommendations; and judicial mandates to SU services. *Need for SU services* was coded as “yes” or “other” (no, legitimate skips, and other missing data). Handling missing data in this way represents a “lower bound” conservative estimate because blank fields could represent a true negative (e.g., not in need of SU services) or true missing (e.g., found to be in need, but not recorded in database) (Dennis et al., 2019). For *level of community supervision*, we categorized the justice status reported by the JJ sites as “more” community supervision (coded 2) if the youth was on probation, parole, or in a juvenile drug treatment court program, “less” (coded 1) if the justice status was other community supervision (e.g., paying a fine or doing community service) or diversion, and a catch-all “other” group (coded 0) for dispositions not involving community supervision, or when no status was indicated.

Contrast coding was used to examine the effects of experimental *Condition* assignment and *TimeEffect* (i.e., study phase) on recidivism, and allowed for the creation of interaction terms between condition and study phase. Analysis using contrast coding tests differences between groups within a dataset by assigning weights to various levels of categorical variables as long as the sum of the weights is zero (Davis, 2010). The *Condition* variable was coded − 1 for Core and + 1 for Enhanced sites. Three variables were created to examine changes in recidivism rates across the four study phases. *TimeEffect1* tests the effect of Pre-randomization (T2 = + 1) compared with the Baseline (T1 = − 1) and ignores later time periods (T3 = 0 and T4 = 0). *TimeEffect2* tests the effect of experiment phase of the study when half of the sites were randomly assigned to the Enhanced condition (T3 = + 1 and T4 = + 1) compared to the Baseline (T1 = − 1) and Pre-randomization (T2 = − 1) phases of the study. *TimeEffect3* tests differences in recidivism between the early experiment phase (T3 = − 1) and the late experiment phase (T4 = + 1) and ignores the first two time periods (T1 = 0 and T2 = 0). Finally, three interaction terms were created: Condition*TimeEffect1, Condition*TimeEffect2, and Condition*TimeEffect3.

### Analytic plan

Data analyses were conducted using IBM SPSS software (Version 28). To test the hypothesis that 1-year recidivism rates will decrease over the course of the study for Core sites and that sites in the Enhanced condition will show greater reductions in recidivism compared to sites in the Core intervention condition, we first conducted a chi-square test of independence for each time period to examine recidivism rates by condition. Next, we conducted a multivariate logistic regression to examine the effects of site, condition, and study phases on recidivism. Since common effect size statistics, such as Cohen’s d, cannot be estimated in a logistic regression model, odds ratios are reported as they can be used as an effect size statistic.

Two logistic regression models were run. In Model 1, we entered *Site*, *need for SU services* and *level of supervision* variables. Model 2 included additional predictor variables: *Condition*, the three study phase contrast variables, and interactions terms for condition with each of the study phase contrasts, as well as *Site*, *need for SU services*, and *level of supervision*. This approach enabled us to examine the predictive value of the study related factors on recidivism beyond site baseline measures.

## Results

### Descriptive statistics

**Fig. 1:**
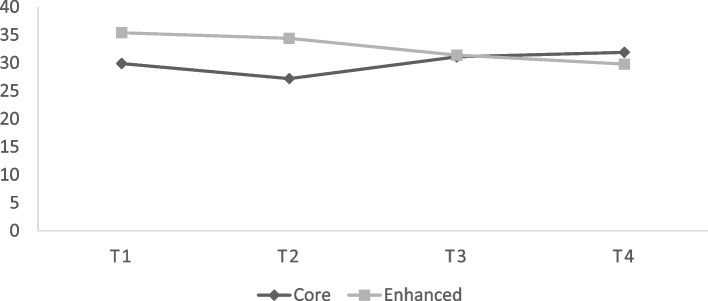
Changes in Recidivism Rates over Time by Experimental Condition

As noted earlier, there were 18,698 youth in the study. The total number of youth who were arrested and/or referred again to the JJ system within 1 year after the initial arrest/referral was 5949 for an overall 1-year recidivism rate of 31.8%. Table 1 displays recidivism rates for each of the four time periods by experimental condition. These data are graphically displayed in Fig. 1. Enhanced sites had significantly (*p* < .05) higher recidivism rates compared with Core sites during the baseline (T1: 35.4% vs. 29.9%) and pre-randomization (T2: 34.4% vs. 27.2%) time periods. By the early experiment period (T3), recidivism rates were essentially the same for Core (31.1%) and Enhanced (31.4%) sites with decreases among Enhanced sites and increases among the Core sites. By the late experiment (T4), the recidivism rate for the Enhanced sites further decreased and were lower (29.8%) than the rate for Core sites (31.9%), a non-statistically significant difference.

**Table 1:** 12-month arrest/referral recidivism by time period and condition

Time	N	Experimental Condition		Χ^**2**^
		Core	Enhanced	
T1- Baseline	6869	29.9	35.4	22.269***
T2- Pre-Randomization	5153	27.2	34.4	30.407***
T3- Early Experiment	4056	31.1	31.4	0.056
T4- Late Experiment	2620	31.9	29.8	1.390

* < 0.1; ** < 0.01; *** < 0.001

In the first logistic regression model, *Site*, *need for SU services*, and *level of supervision* were entered (see Model 1, Table 2). Controlling for the other variables in the model, *Site* was statistically significant (Wald chi-square (19) = 1232.981, *p* <. 001). The recidivism rate for Site20, the reference site, was 22.3%. Sixteen of the 19 sites were significantly different from the reference site. Odds ratios in Model 1 reflect the wide range of differences between sites. For example, youth at Site15 were 88% less likely to recidivate (OR = .121) compared to youth at the Site20 and youth at Site16 were 3.394 times more likely to recidivate compared to youth at the reference site. *Need for SU services* was significantly associated with recidivism such that youth in need of services were almost twice as likely (OR = 1.883) to recidivate than those without an identified need for SU services. Finally, the justice status or disposition of the juvenile’s case was associated with recidivism. Compared to juveniles with other types of disposition, those classified as “less”, that is, those who were diverted or had some other community supervision besides probation were less likely to recidivate (OR = .798) and those placed on formal or more intensive supervision were more likely to recidivate (OR = 1.569).

**Table 2:** Logistic regression model of 1-year recidivism (*N* = 18,698)

	Model 1			Model 2		
Variable	Coefficient	SE	OR	Coefficient	SE	OR
Constant	−1.423***	.047	.287	−1.394***	.102	0.248
Site						
Site1	− 0.996***	.158	0.369	−1.026***	.159	0.358
Site2	−1.819***	.224	0.162	−1.865***	.224	0.155
Site3	−0.373***	.097	0.689	−0.445***	.098	0.641
Site4	0.620***	.124	1.858	0.548***	.126	1.731
Site5	−0.015	.084	0.985	−0.083	.085	0.920
Site6	0.611***	.098	1.842	0.593***	.098	1.809
Site7	0.713***	.122	2.041	0.691***	.122	1.996
Site8	0.690***	.124	1.994	0.606***	.126	1.832
Site9	−0.377*	.172	0.686	−0.407*	.173	0.666
Site10	0.720***	.084	2.054	0.671***	.085	1.957
Site11	0.899***	.075	2.457	0.824***	.077	2.281
Site12	−0.322***	.083	0.725	−0.382***	.085	0.682
Site13	− 0.046	.138	0.955	−0.086	.138	0.918
Site14	0.535***	.065	1.707	0.454***	.068	1.575
Site15	−2.099***	.135	0.123	−2.112***	.135	0.121
Site16	1.223***	.091	3.399	1.222***	.091	3.394
Site17	0.481***	.085	1.618	0.392***	.088	1.481
Site18	0.243**	.094	1.275	0.175	.096	1.191
Site19	−0.153	.094	0858	−0.253**	.097	0.776
SU Need	0.633***	.037	1.883	0.637***	.037	1.890
Level of Supervision (other)						
less	−0.226*	.089	0.798	−0.205*	.090	0.815
more	0.450***	.088	1.569	0.473***	.089	1.605
Condition						
TimeEffect1				−0.020	.022	0.980
TimeEffect2				0.018	.019	1.018
TimeEffect3				0.017	.030	1.017
Condition* TimeEffect1				−0.001	.022	0.999
Condition* TimeEffect2				−0.091***	.019	0.913
Condition* TimeEffect3				−0.035	.030	.965
Model Chi-Square (df)	2520.577 (22)				2545.549 (28)	
Nagelkerke R^2^	.177				.178	

*p < .05, ** *p* < .01, *** *p* < .001

All predictor variables were entered into a second logistic regression model (see Model 2, Table 2). *Site* remained a significant predictor (Wald chi-square (19) = 1219.873, *p* < .001) of recidivism. Inclusion of other predictors from Model 2 resulted in very slight changes in the adjusted odds ratios for *Site*. *Need for SU services* and *level of supervision* remained statistically significant and in the expected direction. *TimeEffect1* tested the impact of Core intervention on recidivism by comparing rates during Pre-randomization (T2) with the Baseline (T1) and was not statistically significantly related to recidivism (b = − 0.020, se = .022, *p* > .05). *TimeEffect2* examined the impact of the Enhanced intervention on recidivism during the experiment phase and compared rates during T3 and T4 with rates during the Baseline (T1) and Pre-randomization (T2) phases of the study and was not statistically significant (b = 0.018, se = .019, *p* > .05). The third variable created to examine changes in recidivism rates over study time periods is *TimeEffect3* which tests differences in recidivism between the early experiment phase (T3) and the late experiment phase (T4) and was not statistically significant (b = 0.017, se = .030, *p* > .05).

The last set of predictors of recidivism were interactions of the experimental condition variable with each of the three *TimeEffect* variables. Of the three, only the interaction of *Condition* by *TimeEffect2* was statistically significant (b = − 0.091, se = .019, *p* < .001) with an OR = .913. Although the estimated effect size was small, an 8.7% reduction in recidivism among 18,698 youth in our sample is equal to 1627 youth.

## Discussion

Identifying SU problems and disorders among adolescents involved with the legal system and linking them to appropriate treatment services are key acts in reducing long-term delinquent behavior and reoffending (Sullivan et al., 2007; Zonnevylle-Bender et al., 2007). The importance of early intervention and continued service delivery to treat substance use disorders is supported by prior research findings that SU problems and delinquent behavior in adolescence are often co-occurring (Ray et al., 2016; Warren et al., 2016), and treatment for SU disorders may reduce behavioral problems (Hoeben et al., 2016; Tanner-Smith et al., 2020). Most JJ agencies do not directly provide SU services instead refer youth to external service providers (Scott et al., 2019), which requires interorganizational communication and collaboration (Welsh et al., 2021; Wasserman et al., 2021). Thus, one of the goals of the JJTRIALS Cooperative was to increase cooperation between juvenile justice and substance use treatment agencies in identifying and addressing substance use service needs of justice-involved youth.

Other JJTRIALS studies have examined the direct effects of Core-only vs Enhanced intervention strategies for improving receipt of SU services along the Behavioral Health Services Cascade. The Belenko et al. (2022) study included 30 sites with data through referral whereas the Knight et al. (2022) study included 20 sites with data covering the full Cascade (the steps following referral are treatment initiation, treatment engagement, and continuing care for 90 days or longer). Referral to treatment among youth identified as in need of SU services increased over time for all sites compared with the baseline period, and the increase was greater in sites receiving external facilitation (Belenko et al., 2022). The second study found that Core-only intervention was effective at increasing service receipt over time relative to baseline (Knight et al., 2022). Although there was no difference between Core-only and Enhanced conditions on receipt of treatment services over time, youth in Enhanced sites began treatment more quickly than those in Core-only and progressed further along the Cascade steps from screening/assessment (step 1) to continuing care (step 6) (Knight et al., 2022). In summary, both studies found that the Core-only set of interventions were effective in increasing referral to SU treatment and increasing treatment initiation. Both studies found some additional, but modest benefits of the structured facilitation.

The present study indirectly assessed the effectiveness of organizational change interventions (i.e., Core-only vs. Enhanced) with JJ and Behavioral Health services agencies on one-year recidivism. One of three hypothesized effects, a condition by time interaction contrasting the Baseline and Pre-randomization phases of the study versus the Early and Late Experiment phases, was statistically significant (b = − 0.091, se = .019, *p* < .001) with an OR = .913. The results indicate youth in the Enhanced condition sites were approximately 9% less likely to recidivate during the experiment phase (T3 and T4) than during the Baseline and Pre-randomization phases compared to youth in Core condition sites. Although the estimated effect size was small, it reflected an estimated reduction in 1627 recidivism events among the 18,698 youth in our sample. Our findings suggest that the expert, manual driven facilitation provided by external facilitators to interagency workgroups at Enhanced sites produced the hypothesized result of lower recidivism in the Enhanced sites relative to the Core-only intervention sites by the end of the study.

This indirect effect underscores the value of organization level interventions on at-risk youth recidivism. Given challenges in collecting, harmonizing, and integrating data collected from sites with differing procedures for recording and storing recidivism data, and likelihood of regression to the mean in recidivism, the experimental effect is, arguably, an underestimate. Furthermore, given the higher costs associated with providing external facilitation and the small magnitude of the direct effect of Enhanced intervention on recidivism, some may regard this facilitation to not be cost effective. Future research should examine the cost-effectiveness of providing all implementation strategies (Core+Enhanced) compared to providing only the Core set of organizational interventions.

### Limitations and future directions

The current study had a few limitations. First, it is important to note that the recidivism measure used in the present study involved tracking youth under community supervision to determine whether they were arrested/referred in the 12 months following an initial arrest/referral. However, we were not able to identify youth who had completed probation when their new arrest or referral occurred. Second, current data were accepted and “cleaned” from case record data in participating jurisdictions. Much data was provided in the form of electronic case records, but some data had to be extracted from probation officer case files. Thus, this method of data collection limited provision of routine data-quality oversight procedures.

An evaluation of specific treatment services in reducing drug use or delinquency was not conducted. Rather, the current study sought to assess the impact of organizational implementation strategies on JJ agencies to change SU screening and referral to treatment practices on youth recidivism. Although lower recidivism among youth in Enhanced condition sites by the end of the study is attributed to Research Center support of interagency change teams in their quality improvement efforts, both youth- and site-level factors were also associated with screening, identification of SUD problems, and referral to treatment among youth at JJ sites (Wasserman et al., 2021; Belenko et al., 2022) as well as treatment initiation, engagement and continuing care (Knight et al., 2022).

It should be noted that there were large site differences in recidivism rates at baseline (Robertson et al., 2020). Although sites were randomly assigned to an intervention condition using a Matched Block design (see Knight et al., 2016), Core-only vs. Enhanced sites differed significantly in recidivism rates at baseline. Many of the factors associated with site differences in referral to treatment rates, such youth race/ethnicity, severity of offense, and level of supervision were also associated with site differences in recidivism. Unfortunately, randomization was not effective in balancing our baseline recidivism risk. Further research is being conducted using qualitative data collected from the Core-only and Enhanced sites to identify other explanations for site differences that would be amenable to organizational intervention. In addition, future research should examine the broad range of factors that influence referral of juveniles with SUD to treatment, how facilitators work with interagency groups to achieve cross system goals, and how agencies change their practices.

## Conclusion

A substantial proportion of youth involved with the legal system experience substance use and behavioral problems (Burke et al., 2015; Choi et al., 2017; Haney-Caron et al., 2019). Active collaboration between juvenile justice and behavioral health agencies is essential for reducing continued contact with the legal system. The results of the present study suggest the potential value of skilled facilitators to assist JJ supervision agencies utilization of data driven, continuum of service practices designed to connect drug involved youth on probation to needed community services. The recidivism effect, although low in magnitude, found in the JJ-TRIALS project provides some indication of the efficacy of these efforts.

## Data Availability

The data that support the findings of this study are available from Justice Community Opioid Innovation Network (JCOIN). Restrictions apply to the availability of these data, which were used under license for the current study, and so are not publicly available. Data are however available from the authors upon reasonable request and with permission of JCOIN.
